# Comparison of outcomes of proximal versus distal and combined splenic artery embolization in the management of blunt splenic injury: a report of 202 cases from a single trauma center

**DOI:** 10.1007/s00464-023-09960-5

**Published:** 2023-03-08

**Authors:** Being-Chuan Lin, Cheng-Hsien Wu, Yon-Cheong Wong, Huan-Wu Chen, Chen-Ju Fu, Chen-Chih Huang, Chen-Te Wu, Yi-Kang Ku, Chien-Cheng Chen, Ting-Wen Sheng, Chun-Bi Chang

**Affiliations:** 1 Division of Trauma & Emergency Surgery, Department of Surgery, Chang Gung Memorial Hospital, Chang Gung University, 5, Fu-Hsing Street, Kwei-Shan, Tao-Yuan City, 333 Taiwan; 2 Division of Emergency and Critical Care Radiology, Department of Medical Imaging and Intervention, Chang Gung Memorial Hospital, Chang Gung University, Tao-Yuan City, Taiwan; 3 Department of Medical Imaging and Intervention, New Taipei Municipal Tucheng Hospital, Chang Gung Medical Foundation, New Taipei City, Taiwan

**Keywords:** Splenic artery embolization, Blunt splenic injury, Pseudoaneurysm, Contrast extravasation, Location of embolization

## Abstract

**Background:**

To compare the outcomes of blunt splenic injuries (BSI) managed with proximal (P) versus distal (D) versus combined (C) splenic artery embolization (SAE).

**Methods:**

This retrospective study included patients with BSI who demonstrated vascular injuries on angiograms and were managed with SAE between 2001 and 2015. The success rate and major complications (Clavien–Dindo classification ≥ III) were compared between the P, D, and C embolizations.

**Results:**

In total, 202 patients were enrolled (P, *n* = 64, 31.7%; D, *n* = 84, 41.6%; C, *n* = 54, 26.7%). The median injury severity score was 25. The median times from injury to SAE were 8.3, 7.0, and 6.6 h for the P, D, and C embolization, respectively. The overall haemostasis success rates were 92.6%, 93.8%, 88.1%, and 98.1% in the P, D, and C embolizations, respectively, with no significant difference (*p* = 0.079). Additionally, the outcomes were not significantly different between the different types of vascular injuries on angiograms or the materials used in the location of embolization. Splenic abscess occurred in six patients (P, *n* = 0; D, *n* = 5; C, *n* = 1), although it occurred more commonly in those who underwent D embolization with no significant difference (*p* = 0.092).

**Conclusions:**

The success rate and major complications of SAE were not significantly different regardless of the location of embolization. The different types of vascular injuries on angiograms and agents used in different embolization locations also did not affect the outcomes.

**Graphical abstract:**

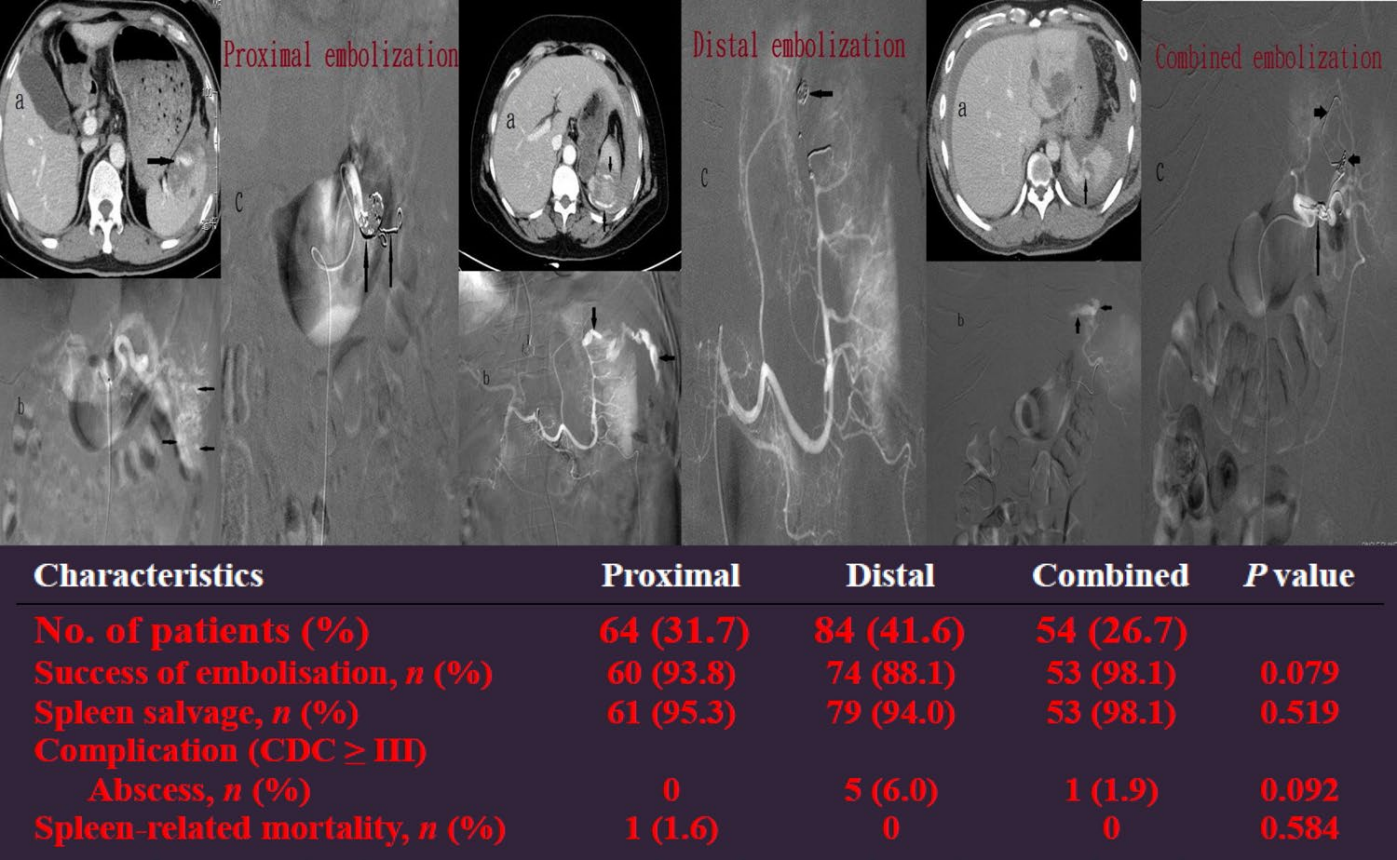

In 1981, Sclafani published the first report of splenic artery embolization (SAE) using gelfoam, steel particles, and vasopressors in three patients with blunt splenic injuries (BSI) as an alternative to surgery [1], and reported a 95.1% spleen salvage rate in 60 patients with BSI using SAE in 1995 [2]. Overtime, the management of BSI has evolved. Nonoperative management (NOM) is the current standard of care for hemodynamically stable patients, and SAE is an integral adjunct to NOM. The splenic artery can be embolized proximally, distally or in combination. However, the location of embolization, its safety, efficacy, and complications remain debatable. For proximal (P) embolization, the goal is to decrease perfusion pressure to achieve hemostasis and the rich network of collateral circulation enters the spleen to decrease the risk of infarction and abscess formation and preserve function [3–6]. For distal (D) embolization, the goal is to focally devascularize the location of the injury while preserving splenic artery patency, which may lead to wedge infarctions or abscess formation [7, 8]. Combined (C) embolization is defined as the combination of both techniques.

## Materials and methods

### Study design and period

This study aimed to compare the clinical success and complications in 202 patients with BSI who demonstrated vascular injuries on angiograms and were managed with either P, D, or C embolisation of the splenic artery**.** Covering a 15-year period from January 1, 2001 to December 31, 2015, data of patients with BSI who underwent angiogram were retrospectively reviewed from the trauma registry and medical records. This study was approved by the Institutional Review Board of Chang Gung Memorial Hospital. The requirement for an informed consent was waived owing to the study design, and the personal information of the patients was kept confidential. As an observational cohort study, the STROBE guidelines were observed [9].

### Study population

**Fig. 1:**
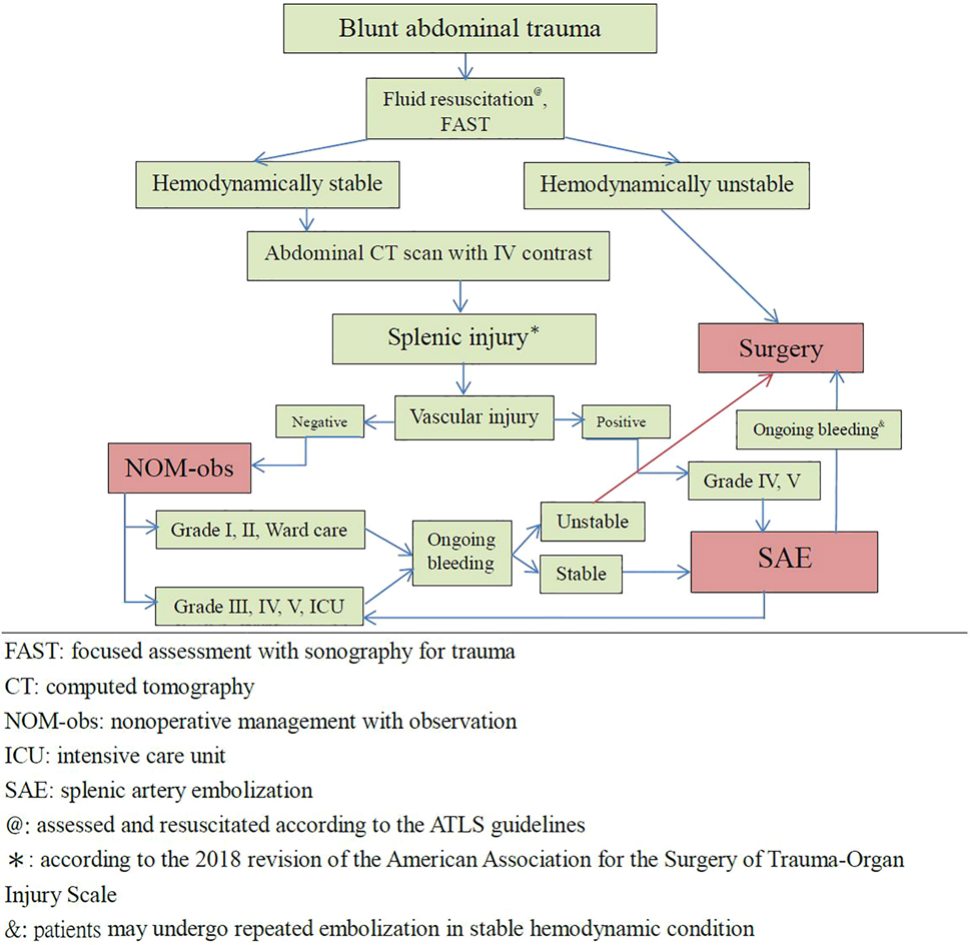
Algorithm for management of patients with blunt splenic injury

Our hospital is a 3704-bed level 1 trauma center in Northern Taiwan with a multidisciplinary collaboration team, including 24/7 in-house year-round attending trauma surgeons and 24/7 in-house interventional radiologists, and the operating room and angiographic suite were available 24 h. In addition to a multidisciplinary teamwork, clinical guidelines for management of BSI have been established and followed (Fig. 1) [10]. Patients with an Abbreviated Injury Score indicating splenic injury who underwent SAE were included in the study, while those without vascular injuries on angiograms were excluded.

### Injury grading and resuscitation

All patients had splenic injuries documented on their admission abdominal computed tomography (CT) scans that were graded and interpreted by trauma surgeons and interventional radiologists. Splenic injuries were graded according to the 2018 American Association for the Surgery of Trauma-Organ Injury Scale (AAST-OIS) revision **(**Table 1**) **[11].

**Table 1:** Spleen organ injury scale—2018 revision

AAST grade	AIS severity	Imaging criteria (computed tomography findings)
I	2	Subcapsular hematoma < 10% surface area
		Parenchymal laceration < 1 cm depth
		Capsular tear
II	2	Subcapsular hematoma 10–50% surface area; intraparenchymal hematoma < 5 cm
		Parenchymal laceration 1–3 cm
III	3	Subcapsular hematoma > 50% surface area; ruptured subcapsular or intraparenchymal hematoma ≥ 5 cm
		Parenchymal laceration > 3 cm depth
IV	4	Any injury in the presence of a splenic vascular injury or active bleeding confined within splenic capsule
		Parenchymal laceration involving segmental or hilar vessels producing > 25% devascularization
V	5	Any injury in the presence of splenic vascular injury with active bleeding extending beyond the spleen into the peritoneum
		Shattered spleen

Vascular injury is defined as a pseudoaneurysm or arteriovenous fistula and appears as a focal collection of vascular contrast that decreases in attenuation with delayed imaging. Active bleeding from a vascular injury presents as vascular contrast, focal or diffuse, that increases in size or attenuation in delayed phase. Vascular thrombosis can lead to organ infarction. Grade based on highest grade assessment made on imaging, at operation or on pathologic specimen. More than one grade of splenic injury may be present and should be classified by the higher grade of injury. Advance one grade for multiple injuries up to a grade III

AAST: American Association for the Surgery of Trauma

AIS: Abbreviated Injury Scale

### Management of protocol

Patients with BSI who are hypotensive and refractory to resuscitation require surgery. All hemodynamically stable patients (including those with shock at triage and response to resuscitation) with grade I or II splenic injuries on admission abdominal CT scans were simply observed in the ward. Patients with grades III, IV, and V without vascular injuries on abdominal CT scans underwent NOM-obs in the intensive-care unit. Patients with vascular injuries on abdominal CT scans were considered for SAE (Fig. 1) [10].

### SAE technique

**Fig. 2:**
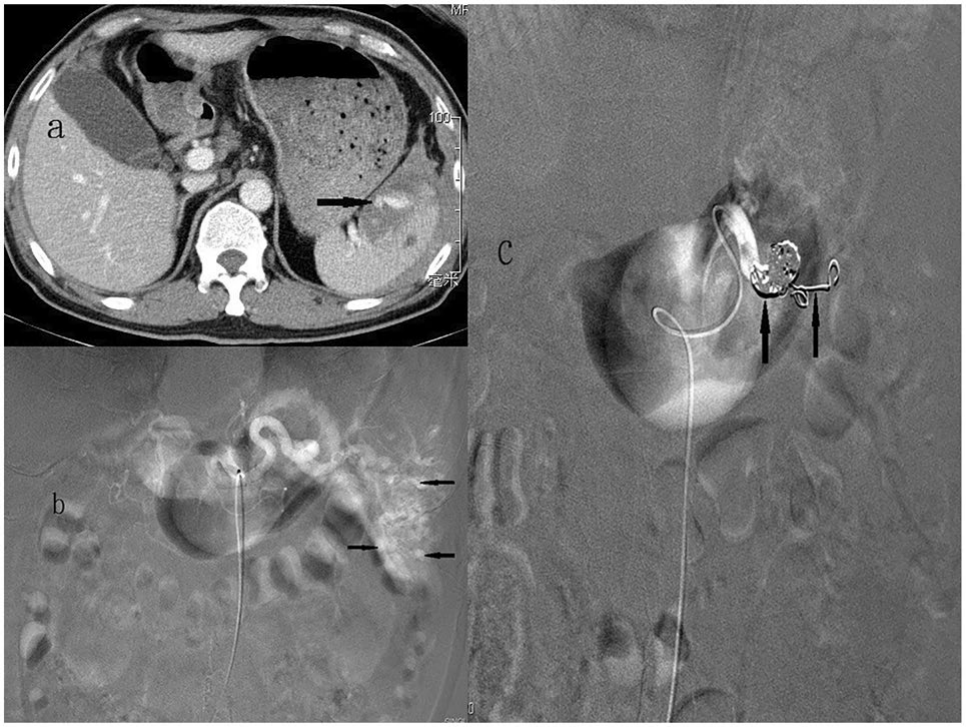
58-Year-old man with a grade IV blunt splenic injury underwent proximal splenic arterial embolization. **a** Admission contrast-enhanced helical computed tomography scan demonstrates a low attenuation laceration with a big pseudoaneurysm (arrow) and perisplenic hematoma. **b** The splenic angiogram demonstrates multiple pseudoaneurysms (arrows) at lower half of spleen. **c** The post-embolization angiogram demonstrates the deployment of metallic coils in the main splenic artery (arrows). Pseudoaneurysm is no longer present

**Fig. 3:**
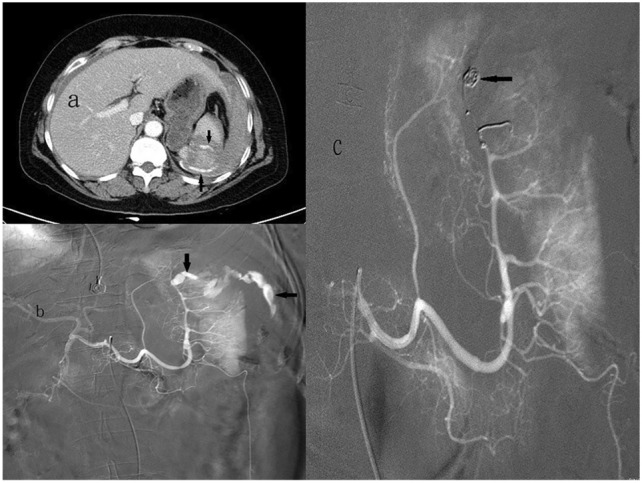
61-Year-old woman with a grade V blunt splenic injury underwent distal splenic arterial embolization. **a** Admission contrast-enhanced helical computed tomography scan demonstrates splenic laceration with perisplenic contrast medium extravasation (arrows). **b** The splenic angiogram demonstrates an active leak of contrast medium into the parenchyma with peritoneal cavity extension (arrows) from a branch artery of upper pole. **c** Metallic coils (arrow) were deployed selectively at upper pole. The post-embolization angiogram demonstrates no further extravasation from the injured spleen

**Fig. 4:**
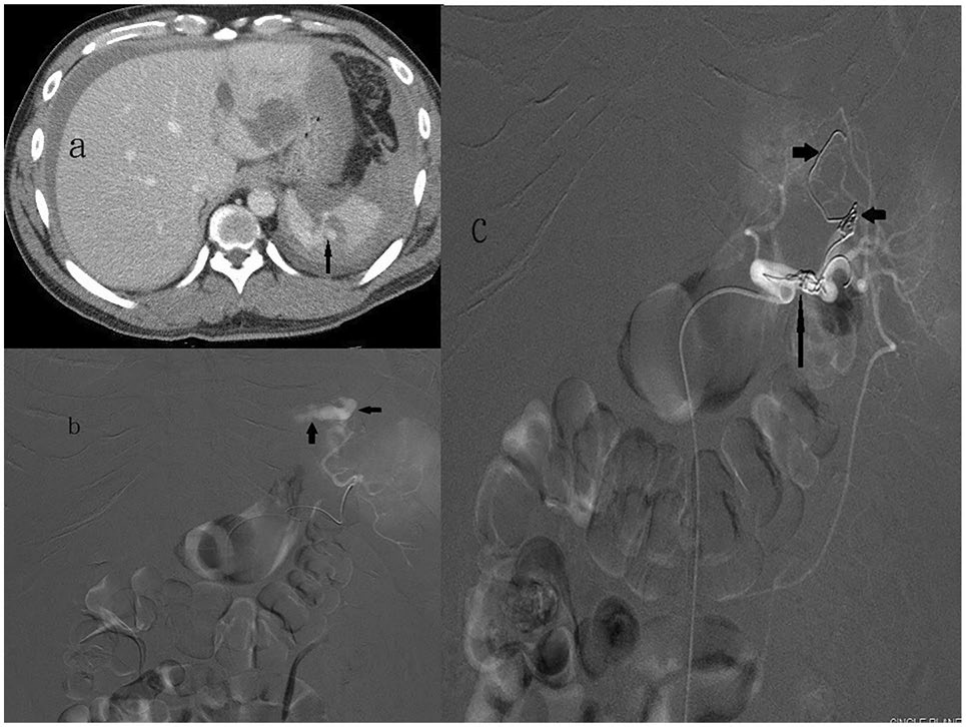
32-Year-old man with a grade IV blunt splenic injury underwent combined splenic arterial embolization. **a** Admission contrast-enhanced helical computed tomography scan demonstrates a low attenuation laceration with a pseudoaneurysm (arrow) and perisplenic hematoma. **b** The splenic angiogram demonstrates an active leak of contrast medium from a branch artery of upper pole (arrows). **c** The post-embolization angiogram demonstrates combined embolization with coils in the proximal splenic artery (arrow) and in the distal branch (arrowheads)

Femoral artery access was obtained by eventual placement of a 4- or 5-French catheter into the celiac trunk. Celiac angiogram was performed to demonstrate not only the splenic artery anatomy but also the collaterals to the spleen, including the dorsal and great pancreatic arteries, and to identify active contrast extravasation, pseudoaneurysm, degree of devitalised spleen, and abnormally truncated vessels. For proximal embolization (Fig. 2), a coil pack is typically placed precisely distal to the main pancreatic artery branches to allow for continued blood supply via the collateral vessels [12, 13], the dorsal pancreatic artery and the pancreatica magna should be identified on angiograms, and embolization is performed between these two branches to avoid devascularization of the pancreas and ischemic pancreatitis. For distal embolization (Fig. 3), the microcatheter is placed as distally as possible in the injured splenic artery branches before embolization to completely preserve the spleen [14]. Combined embolization (Fig. 4) was defined as a combination of both techniques. Various agents, such as coils or absorbable 1–3 mm gelfoam cubes (Upjohn, Inc., Kalamazoo, MI, USA), can be used either alone or in combination. The goal of embolization is to achieve cessation of contrast medium extravasation or total obliteration of the pseudoaneurysm.

### Definition

A pseudoaneurysm was defined as a contrast medium confined within the splenic capsule. Contrast extravasation was defined as the leakage of the injected contrast medium extending beyond the spleen into the peritoneum. Success of the SAE was defined as achieving hemostasis after the first embolization. Spleen salvage was defined as patient discharge with the spleen in situ. Complications were graded as minor or major according to the Clavien–Dindo classification (CDC) [15]. Grade I and II complications that did not require treatment were classified as minor. Grade III, IV, and V complications requiring endoscopic or surgical treatment that were life-threatening or resulted in death were classified as major [15]. In this study, major complications (CDC ≥ III) between the locations of embolization were compared, and asymptomatic splenic infarcts were not included. Spleen-related mortality was defined as death due to persistent bleeding from a splenic injury.

### Demographic data analysis

Medical charts were reviewed retrospectively for age, sex, trauma mechanism, injury severity score (ISS), imaging study, location of embolization, and outcomes. Patients were divided into three groups depending on the location of embolization as follows: P, D, and C embolization.

### Statistics

Categorical data are presented as numerical values and continuous data as median (i.q.r.) values. The Mann–Whitney *U* test was used for continuous data. For comparisons of categorical variables, Pearson’s chi-squared test or Fisher's exact test was used, as appropriate. All statistical analyses were performed using the SPSS® version 20.0 (IBM, Armonk, New York, USA). Significance was set at *P* < 0.05 (two‐sided).

## Results

### Characteristics of the study population and management

**Fig. 5:**
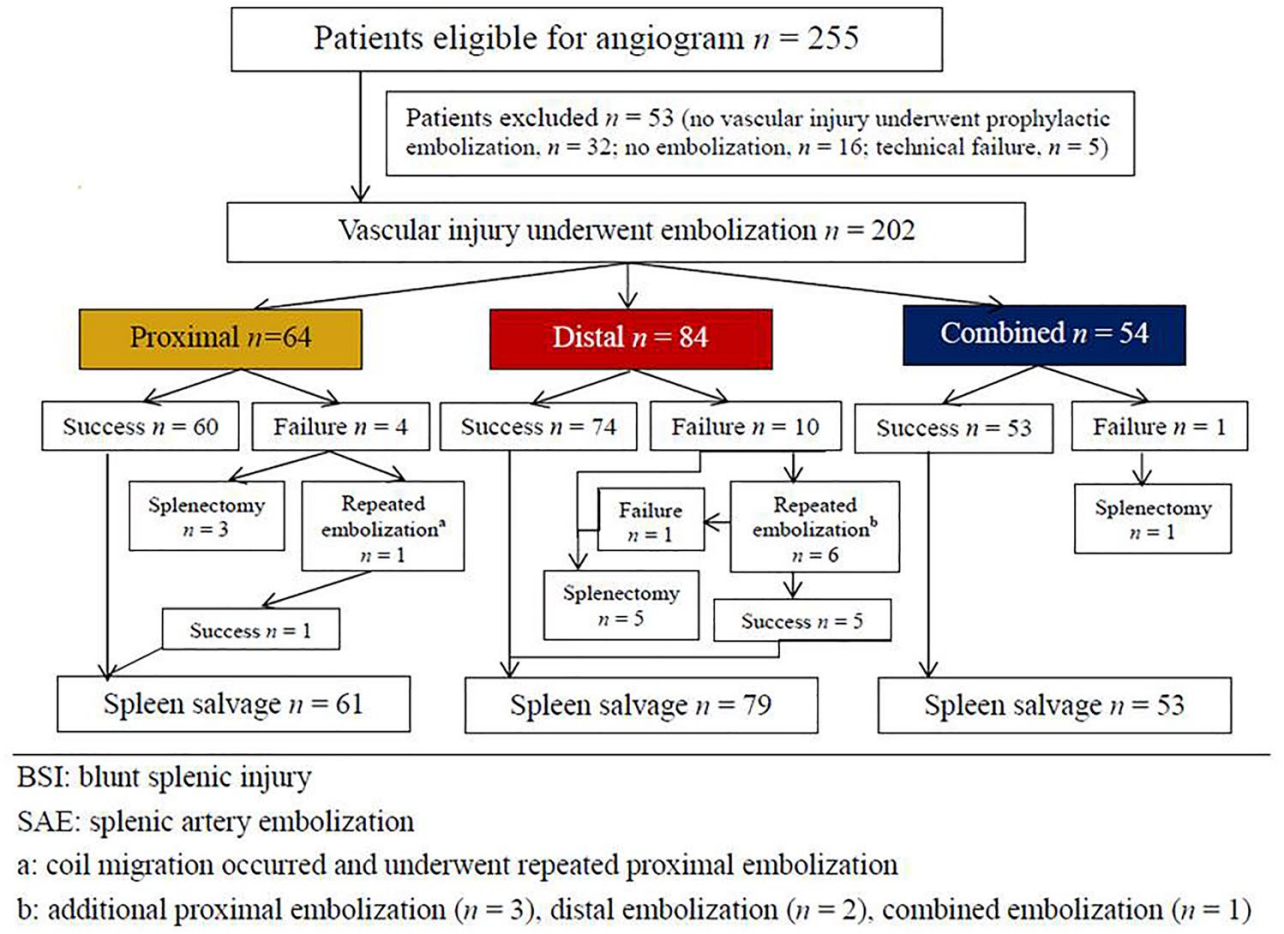
Flow diagram of patients with BSI who demonstrated vascular injury on angiogram and underwent SAE in Chang Gung Memorial Hospital from 2001 to 2015

During the study period, 255 patients who underwent angiogram and 53 patients with no vascular injuries with prophylactic embolization (*n* = 32), no vascular injury without embolization (*n* = 16), and experienced technical failure (*n* = 5) were excluded from the study. The remaining 202 patients who demonstrated vascular injuries on angiograms after complete embolization were included in the study (P, *n* = 64; D, *n* = 84; C, *n* = 54) (Fig. 5). Baseline characteristics at admission to the emergency department are presented in Table 2. In total, 166 patients were male, and 36 were female, with a median age of 39.5, 37.5, and 32 years in the P, D, and C embolization groups, respectively (*p* = 0.544). The median OIS score was 4 for the P, D, and C embolization groups (*p* = 0.350), and the median ISS was 25 for the P, D, and C embolization groups (*p* = 0.528). The median time from injury to embolization was 8.3, 7.0, and 6.6 h in the P, D, and C embolization groups, respectively (*p* = 0.548) (Table 2). No significant differences in sex, age, shock at triage, initial serum hemoglobin level, severity of injuries, length of stay, and time from injury to embolization were observed among the three groups (Table 2). In this series, 187 patients achieved hemostasis after SAE with an overall success rate of 92.6%, and 93.8%, 88.1%, and 98.1% for the P, D, and C embolization groups, respectively, although the difference was not significant (*p* = 0.079) (Table 3). Pseudoaneurysms were the most common vascular injuries on angiograms in 128 patients (63.4%), and the success rates were 92.8%, 90.9%, and 96.8% for the P, C, and C embolization groups, respectively, which were not significantly different (*p* = 0.593) (Table 4). The correlation between the success rate of other vascular injuries and embolization location is presented in Table 4. Coils or microcoils alone were the most commonly used embolizers in 167 patients (82.7%), and the success rates were 94.0%, 87.9%, and 98.0% for P, D, and C embolizations, respectively, and were not significantly different (*p* = 0.112) (Table 5). Table 5 presents the success rates of the other embolizers according to the location of embolization. Major complications (CDC III) such as post-embolization splenic abscess that required further intervention occurred in six patients, with no significant difference among the three groups (*p* = 0.092) (Table 3). Five patients died after SAE, with an overall mortality rate of 2.5%, and four of them died of head cause; the other patient died of splenic rebleeding despite receiving emergent surgery, with a spleen-related mortality rate of 0.5% (Table 3).

**Table 2:** Demographic data and clinical characteristics of the proximal, distal, and combined embolizations in 202 patients with BSI who demonstrated vascular injury on angiography

Characteristics	Proximal	Distal	Combined^a^	*P* value
No. of patients (%)	64 (31.7)	84 (41.6)	54 (26.7)	
Gender				
Male, *n* (%)	54 (84.4)	71 (84.5)	41 (75.9)	0.374
Female, *n* (%)	10 (15.6)	13 (15.5)	13 (24.1)	
Age (years), median (Q1, Q3)	39.5 (25, 50)	37.5 (21.3, 57.8)	32 (22.8, 46.3)	0.544
Shock at triage, *n* (%)	18 (28.1)	14 (16.7)	9 (16.7)	0.170
Systolic blood pressure (mmHg), median (Q1, Q3)	114 (94, 138)	117 (94, 132)	111 (97, 132)	0.991
Initial serum hemoglobin (g/dL), median (Q1, Q3)	11.2 (9.1, 13.6)	11.3 (9.4, 13.4)	115 (9.4, 13.2)	0.911
Grade of splenic injury				0.708
II, *n* (%)	1 (1.6)	2 (2.4)	0	
2018 AAST-OIS III, *n* (%)	5 (7.8)	7 (8.3)	2 (3.7)	
IV, *n* (%)	27 (42.2)	42 (50.0)	26 (48.1)	
V, *n* (%)	31 (48.4)	33 (39.3)	26 (48.1)	
OIS, median (Q1, Q3)	4 (4, 4)	4 (4, 5)	4 (4, 5)	0.350
Coexistent injuries (%)	47 (73.4)	56 (66.7)	33 (61.1)	0.359
Injury severity score, median (Q1, Q3)	25 (20, 34)	25 (16, 29)	25 (19.5, 27.5)	0.528
Length of stay (days), median (Q1, Q3)	9.5 (7, 13)	10 (8, 15)	9 (6, 15)	0.271
Time from injury to SAE (hours), median (Q1, Q3)	8.3 (5.1, 19.3)	7 (5, 57.8)	6.6 (5, 21)	0.548

*BSI* blunt splenic injury, SAE: splenic artery embolization

2018 AAST-OIS: 2018 revision of the American Association for the Surgery of Trauma-Organ Injury Scale

^a^Combination of proximal and distal embolization

**Table 3:** Comparison of outcomes between the proximal, distal, and combined embolizations in 202 patients with BSI who demonstrated vascular injuries on angiography

Characteristics	Proximal	Distal	Combined^a^	*P* value
No. of patients (%)	64 (31.7)	84 (41.6)	54 (26.7)	
Success of embolisation, *n* (%)	60 (93.8)	74 (88.1)	53 (98.1)	0.079
Spleen salvage, *n* (%)	61 (95.3)	79 (94.0)	53 (98.1)	0.519
Complication (CDC ≥ III)				
Abscess,* n* (%)	0	5 (6.0)	1 (1.9)	0.092
Spleen-related mortality, *n* (%)	1 (1.6)	0	0	0.584

*BSI* blunt splenic injury, *CDC* Clavien–Dindo classifications

^a^Combination of proximal and distal embolization

**Table 4:** Comparison of the success rate between the proximal, distal, and combined embolizations in 202 patients with BSI of different vascular injuries on angiogram

Vascular injuries, no. of patients	Location, no. of patients			
	Proximal, *n* = 64	Distal, *n* = 84	Combined^a^, *n* = 54	*p* value
	No. of success/no. of embolization (%)			
Pseudoaneurysm, *n* = 128	39/42 (92.8)	50/55 (90.9)	30/31 (96.8)	*p* = 0.593
Combined^b^, *n* = 36	9/10 (90)	8/10 (80)	16/16 (100)	*p* = 0.195
Contrast extravasation, *n* = 35	11/11 (100)	16/18 (88.9)	6/6 (100)	*p* = 0.367
Arteriovenous fistula, *n* = 3	1/1 (100)	1/1 (100)	1/1 (100)	*p* = 0.223
Total, *n* = 202	60/64 (93.8)	74/84 (88.1)	53/54 (98.1)	*p* = 0.079

*BSI* blunt splenic injury

^a^Combination of proximal and distal embolization

^b^Combined contrast extravasation with pseudoaneurysm

**Table 5:** Comparison of success rate between the proximal, distal, and combined embolizations in 202 patients with BSI with different embolizer

Embolizer, No of patients	Location, no. of patients			
	Proximal, *n* = 64	Distal, *n* = 84	Combined^a^, *n* = 54	*p* value
	No. of success/no. of embolization (%)			
Coil, *n* = 167	47/50 (94.0)	58/66 (87.9)	50/51 (98.0)	*p* = 0.112
Gelfoam, *n* = 12	4/4 (100.0)	6/8 (75.0)	–	*p* = 0.515
Combined coil and gelfoam, *n* = 23	9/10 (90.0)	10/10 (100)	3/3 (100)	*p* > 0.999

*BSI* blunt splenic injury

^a^Combination of proximal and distal embolization

## Discussion

Currently, a higher success rate of SAE of 90% has been reported [2, 10, 14, 16]. The splenic artery can be embolized proximally, distally or in combination. P embolization is the most common choice and is preferred in some reports [2, 6, 12, 14, 17], D embolization in others [18–20], and C embolization in some series [7, 8, 14, 17, 21, 22]. P embolization has a shorter procedural time and presents with a quicker option for providing treatment in multiple splenic injuries (Fig. 2), even with significant anatomic variation and/or vasospasm [14, 16, 23]. However, P embolization can lead to permanent occlusion of the splenic artery and precludes further angiographic management if rebleeding occurs. Compared to P embolization, D embolization is more technically challenging, and it is beneficial in patients with focal, angiographically evident injury and in minimizing global splenic ischemia (Fig. 3). However, segmental bleeding vessels may be overlooked because of vasospasms caused by haematomas, potentially increasing the risk of rebleeding [4, 8, 14, 18]. Combined embolization is reserved for vascular lesions combined with high-grade splenic injuries, and for some vascular injuries, it may not be visible on the initial angiogram and may lead to delayed bleeding once the vasospasm subsides [5, 21, 22, 24]. Reports over the past few years have mainly discussed conflicting clinical outcomes of P versus D embolization, and the optimal location of embolization remains inconclusive [5, 8, 12, 14, 16, 17]. Reports simultaneously comparing the outcomes of P, D, and C embolizations are scant [21, 22]. Our data are the largest series of SAE collected to date from a single center, and we graded our patients using the 2018 AAST-OIS which incorporates vascular injury that facilitates recognition of grade IV and V injuries and is superior to the 1994 classification [10]. Moreover, patients who demonstrated no vascular injuries on angiograms and underwent prophylactic embolization were excluded to minimize bias as much as possible and to demonstrate the outcomes of different locations of embolization. In this report, we analyze and discuss two parts: first, the comparison of P, D, and C embolizations in terms of safety and efficacy of hemostasis and, second, the major complications requiring intervention (CDC ≥ III) of these three embolizations. A meta-analysis of 876 patients from 1991 to 2013 by Rong et al. [16] has revealed the following: P embolization was performed more often than distal embolization and combined significantly (52.1% vs. 24.8% vs. 5.5%, respectively); the overall success rate of SAE was 90.1% (range 72.7–100%); and success rate and embolization location (*P* > 0.05) had no evident association. Conversely, distal embolization was the most common choice in our series (*n* = 84, 41.6%), followed by P embolization (*n* = 64, 31.7%) and combined embolization (*n* = 54, 26.7%) (Table 2). Fifteen patients (P, *n* = 4; D, *n* = 10; C, *n* = 1) with persistent bleeding in our series failed to undergo SAE; the success rate was 92.6% overall, and 93.8%, 88.1%, and 98.1% in the P, D, and C embolization groups, respectively; however, the differences were not significant (*p* = 0.079) (Table 3). One patient who underwent P embolization initially and experienced rebleeding with sudden hemodynamic collapse at the ward 9 days later and died despite receiving emergent surgery. However, no significant difference in mortality was observed between the embolization locations (*p* = 0.584) (Table 3). Of the 15 patients who had failed initial SAE with persistent bleeding, nine (P, *n* = 3; D, *n* = 5; C, *n* = 1) underwent splenectomy, and the other six (P, *n* = 1; D, *n* = 5) achieved hemostasis after a repeat embolization with an overall salvage rate of 95.5%, and of 95.3%, 94.0%, and 98.1% in the P, D, and C embolizations, respectively; the differences were not significant (*p* = 0.519) (Table 3). These data confirm that no significant difference exists between the success rate and the location of embolization, which is in accordance with a previous review [16]. In addition to comparing the success according to the location of embolization, we compared the efficacy of hemostasis in different vascular injuries on angiograms with the location of embolization, which has not been previously reported. In this series, pseudoaneurysm was the most common vascular injury demonstrated on angiograms (*n* = 128, 63.4%), followed by combined contrast extravasation with pseudoaneurysm (*n* = 36, 17.8%) and contrast extravasation (*n* = 35, 17.3%) (Table 4). Based on our data, despite the various types of vascular injuries on angiograms, the success rate was not significantly different according to the location of embolization (Table 4). Coil, the most commonly used material for embolization, can be deposited more accurately, although it may cause migration when undersized to the vessel and rebleeding or more infraction of the spleen [8, 14, 18]. Meanwhile, gelfoam is a water-insoluble, nonelastic hemostatic agent that slows blood flow, hastens thrombus formation, and provides temporary vessel occlusion, allowing recanalization within days or a few weeks [4, 25]. At present, the correlation between the embolization material and severe complications remains controversial. Using resorbable agents reportedly have a higher risk of rebleeding and a higher severe complication rate than using coil [16, 17, 21]. However, other studies have suggested that the difference was not significant [14, 19]. In our study, 167 patients (82.7%) underwent coil embolization, 12 patients (5.9%) underwent gelfoam embolization, and 23 patients (11.4%) underwent combined coil and gelfoam embolization (Table 5). Our data revealed that the success rate of embolization was independent of the embolic material and location of embolization (Table 5). Except for rebleeding, other complications of SAE include splenic infarct, cyst, abscess, and contrast-induced renal insufficiency [8, 16]. In a study by Ekeh et al. [8], the significant complication rate remained higher in individuals who underwent distal embolization than in those who underwent P embolization (22% vs. 4%; *p* = 0.02). Splenic infarction is the most common post-embolization complication; however, the vast majority of these patients are asymptomatic and can be managed nonoperatively [14, 16]. In our protocol, an abdominal CT scan was not routinely performed after SAE unless indicated in selected cases; therefore, data on asymptomatic splenic infarcts are incomplete. In Rong et al.’s review [16], although P embolization had fewer CDC III complications than distal embolization (9.9% vs. 20.0%) or combined embolization (10.8% vs. 23.1%), the *p* value were 0.07 (P vs. D) and 0.16 (P vs. C), respectively, with no significant differences. Reportedly, splenic abscess formation after SAE occurs in 3.8–7% of patients [8, 16]. In our series, splenic abscess after SAE occurred in six patients (3.0%); five of these underwent CT-guided drainage, and the other underwent surgical drainage. Although splenic abscess occurred more commonly in the distal embolization group (*n* = 5), no significant difference was observed among the three groups (*p* = 0.092) (Table 3).

This study had some limitations. First, as this was a retrospective study, the subsequent availability of data is limited to those that were present in the patient’s medical records. Prospective studies are needed to determine the optimal location of embolization for the management of BSI. Second, as this was a single-center study, the internal structure of the trauma and interventional radiology teams may not necessarily reflect that of other hospitals.

## Conclusions

The clinical success rate of SAE was 92.6%, and the splenic salvage rate was 95.5%, which confirmed that SAE for BSI was associated with good success rates. Irrespective of P, D, or C embolization, SAE are effective for hemorrhage control. There was also no statistically significant difference between the location of embolization and major complications.

The different types of vascular injuries on angiograms and embolic agents used also did not impact the outcomes, regardless of the location of embolization.
